# Evaluating the potential of a novel oral lesion exudate collection method coupled with mass spectrometry-based proteomics for oral cancer biomarker discovery

**DOI:** 10.1186/1559-0275-8-13

**Published:** 2011-09-13

**Authors:** Joel A Kooren, Nelson L Rhodus, Chuanning Tang, Pratik D Jagtap, Bryan J Horrigan, Timothy J Griffin

**Affiliations:** 1 Department of Biochemistry, Molecular Biology, and Biophysics, University of Minnesota, 321 Church St SE, 6-155 Jackson Hall, Minneapolis, Minnesota, 55455, USA; 2 Oral Medicine, Diagnosis and Radiology, School of Dentistry, University of Minnesota, 515 Delaware St SE, Minneapolis, Minnesota, 55455, USA; 3 Minnesota Supercomputing Institute, University of Minnesota, 117 Pleasant Street SE, Minneapolis, Minnesota, 55455, USA

**Keywords:** Oral Pre-Malignant Lesion (OPML), Oral Squamous Cell Carcinoma (OSCC), exudate, mass spectrometry-based proteomics, biomarker

## Abstract

**Introduction:**

Early diagnosis of Oral Squamous Cell Carcinoma (OSCC) increases the survival rate of oral cancer. For early diagnosis, molecular biomarkers contained in samples collected non-invasively and directly from at-risk oral premalignant lesions (OPMLs) would be ideal.

**Methods:**

In this pilot study we evaluated the potential of a novel method using commercial PerioPaper absorbent strips for non-invasive collection of oral lesion exudate material coupled with mass spectrometry-based proteomics for oral cancer biomarker discovery.

**Results:**

Our evaluation focused on three core issues. First, using an "on-strip" processing method, we found that protein can be isolated from exudate samples in amounts compatible with large-scale mass spectrometry-based proteomic analysis. Second, we found that the OPML exudate proteome was distinct from that of whole saliva, while being similar to the OPML epithelial cell proteome, demonstrating the fidelity of our exudate collection method. Third, in a proof-of-principle study, we identified numerous, inflammation-associated proteins showing an expected increase in abundance in OPML exudates compared to healthy oral tissue exudates. These results demonstrate the feasibility of identifying differentially abundant proteins from exudate samples, which is essential for biomarker discovery studies.

**Conclusions:**

Collectively, our findings demonstrate that our exudate collection method coupled with mass spectrometry-based proteomics has great potential for transforming OSCC biomarker discovery and clinical diagnostics assay development.

## Background

Oral cancer occurs most commonly (~90%) in the form of oral squamous cell carcinoma (OSCC) and develops in stages starting with healthy oral epithelium progressing to an Oral Pre-Malignant Lesion (OPML) and on to OSCC. The survival rate of OSCC has remained static over the last 30 years at about 50%. However, where malignancy is detected soon after the transition from OPML, treatments are more effective and survival is as high as 80% [1]. Despite the clinical need to distinguish between OSCC and OPML, lesion types are not readily classified by simple visible inspection and more invasive tests are used instead. Currently the gold standard for classifying lesions is to use an incisional biopsy coupled with histological analysis [2,3]. Yet biopsies have numerous limitations: being invasive clinicians are hesitant to perform them, and patients are hesitant to agree to them due to the pain and discomfort of the procedure; the following histology requires expert analysis and is therefore expensive; and issues such as under-sampling can lead to misdiagnosis [4].

An ideal alternative to scalpel biopsy would be a non-invasively collected sample rich in molecular biomarkers which distinguish OPML and OSCC, and potentially predict the transition from pre-malignancy to malignancy. One such alternative is the use of protein or nucleic acid biomarkers in saliva that are secreted or shed from the oral lesions [5,6]. However, despite its benefits, whole saliva is not the direct source of potential biomarkers, and the complexity of the fluid [7] makes identification of potential biomarkers challenging. In contrast to whole saliva, some have directly analyzed incisional biopsy tissues [8,9], but for clinical diagnostics this approach suffers from the same limitations described above for scalpel biopsy.

Given the ongoing need for improved oral cancer detection, we describe here a promising alternative method for the direct and non-invasive sampling of oral lesions, which can be coupled with mass spectrometry (MS)-based proteomics. Our method uses commercially available PerioPaper Strips, traditionally used for oral fluid sampling relevant to periodontal disease [10,11], to directly collect oral lesion exudate. Exudate is defined as the fluid and cellular material present on the surface of inflamed tissue [12]. Our results show that the exudate samples contain ample protein for large-scale proteomics analysis, and that the exudate proteome of OPMLs is distinct from whole saliva, while being highly similar to the proteome of lesion-associated epithelial cells. We also undertook a pilot study comparing healthy tissue and OPML exudates, demonstrating that the method is amenable to quantitative proteomic analysis necessary for biomarker discovery studies. Collectively our results demonstrate the great potential of our exudate collection method for oral cancer biomarker discovery and clinical diagnostics.

## Methods

### Patient information

Exudates and brush biopsies were collected from three patients diagnosed with a dysplastic OPML, at the University of Minnesota Dental School. For each OPML patient, exudate samples were first collected from the oral lesion, followed by collection of the brush biopsy from the same lesion. Exudates and brush biopsies from buccal mucosa, and whole saliva were also collected from three healthy volunteers. The healthy volunteers had no major risk factors for OSCC (non-smokers, moderate to low alcohol use) and were free of oral lesions. All samples were collected with written consent using an IRB protocol approved by the University of Minnesota. Three different lesion and three healthy samples were analyzed to provide some statistical significance for measurements of differential protein abundance between tissue types while balancing the time and cost of large-scale proteomic analysis of individual patient samples.

### Exudate sample collection and protein processing

To collect the exudate we first used rolled cotton to swab away ambient saliva around tissue to be sampled (e.g. OPML). The rolled cotton was then moved adjacent to the area to be sampled to block flow of additional saliva onto the tissue. A PerioPaper strip (Oraflow, Smithtown, New York) was left in position for ~30 seconds. Immediately after collection the strip was placed in a microcentrifuge tube, on ice, and then transferred to a -20°freezer within minutes. The PerioPaper strips were subjected to on-strip trypsin digestion in which each PerioPaper strip containing exudate was submerged in 100 μl buffer containing 5 mM DTT, 100 mM Tris pH 8.0 and boiled for 5 min. After cooling to room temperature, 2 μg of sequencing grade trypsin (Promega, Madison, WI) was added and the microcentrifuge tube was placed in 37°C water bath to digest for 12 hours. The protein digest was then purified and concentrated using Waters Sep-Pak 3cc cartridges as described [6] drying the purified peptides by vacuum centrifuge (~2 hrs or until dry). The peptides were analyzed either directly by mass spectrometry or subjected to strong cation exchange (SCX) HPLC fractionation as described below.

### Brush Biopsy sample collection and protein processing

To collect brush biopsies we first dried the tissue to be sampled as with exudate collection. Next, we collected transepithelial cells from the tissue using an OralCDx brush test kit (OralCDx laboratories, Inc. Suffern, NY) and following manufacturer's suggested procedure. After collection of cells, the brush head was cut off from the handle and submerged in 250 μL of 2× SDS cell lysis buffer (4% SDS, 20% glycerol, 10% 2-mercaptoethanol, and 100 mM Tris-HCl pH 6.8) and 1× protease inhibitors (Complete Mini, Roche Applied Science, Indianapolis, IN, USA) in a 2 mL microcentrifuge tube. In order to extract proteins from the cell lysate while removing detergents and minimizing other impurities, the proteins were precipitated with acetone added at a 5:1 ratio and left overnight at -20°C. Precipitated protein was centrifuged at 6000 rpm for 10 min at 4°C, then rinsed and re-centrifuged with pure acetone twice. Proteins were redissolved in trypsin digestion buffer, quantified using the BCA protein assay (Pierce, Rockford, IL, USA), and digested with trypsin as described above for the exudate samples.

### Collection and processing of control whole saliva samples

To collect the PerioPaper saliva samples we placed the PerioPaper strip at a location in a healthy volunteer's oral cavity where ambient saliva had pooled (the back of the lower lip). The strip was allowed to saturate with saliva (< 20 sec) before being removed. Once collected the PerioPaper saliva samples were immediately placed on ice. On-strip digestion, as described above, was implemented within several minutes of sample collection.

### SCX HPLC fractionation

Peptide digests from exudates, brush biopsies, and the whole saliva samples were subjected to offline SCX HPLC fractionation essentially as in previous studies [7]. A UV chromatogram (215 nm and 280 nm absorbance) was generated for every different sample. For all samples (exudates or whole saliva), SCX fractions containing UV signals indicating the presence of peptides were combined into 9 fractions for subsequent analysis by mass spectrometry. Loading amounts from each peptide fraction were normalized between different samples based on UV absorbance units, to ensure loading of relatively equal amounts of peptides across all different samples being compared.

### Shotgun proteomics analysis: Tandem mass spectrometric analysis and sequence database searching

The overall workflow used for data analysis is shown in Additional File 1, Figure S1. Peptide mixtures from all sample types (exudates, brush biopsy and whole saliva) were analyzed using online capillary liquid chromatography coupled with tandem mass spectrometry (MS/MS) using an LTQ-Orbitrap XL mass spectrometer (Thermo Scientific, San Jose, CA). The chromatography conditions and instrumental parameters used have been described [7]. The .RAW files generated by the LTQ-Orbitrap XL were converted to .MSM file format peaklists using "Quant" module from MaxQuant's (v 1.0.13.13, Max-Planck Institute for Mass Spectrometry, Martinsried, Germany [13,14]. The .MSM files are peaklists with high precursor mass accuracy and limited product ion 'noise' peaks. MaxQuant achieves high precursor mass accuracy by using information from LC-MS precursor peaks. Top 6 MS/MS peaks per 100 Da are selected to generate peaklists with limited background noise peaks. The .MSM peaklists were searched using Mascot (v2.1, Matrix Sciences, London, United Kingdom) Daemon and with following parameters: Orbitrap/FT as the instrument, no SILAC labeling, Methionine oxidation as the only variable modification, Carbidomethyl as the fixed modification, Trypsin as the enzyme, two missed cleavages, MS tolerance at 7 ppm and MS/MS tolerance at 0.5 Da and searched against target-decoy version of Human IPI database (v3.52, Nov 2008) plus contaminant proteins (148372 forward plus reversed sequences). Mascot search generates an output in .dat format that contains peptide-spectrum matching information.

Mascot output .dat files were subjected to statistical validation and protein inference using Scaffold Q+ v 3.0 (Proteome Software, Portland, OR). For peptide identification the false discovery rate threshold was maintained at 1%. For quantification using spectral counts, total spectra identified in a dataset were normalized with spectra identified in dataset that was to be compared. This normalization, which is achieved by using a display option called "Quantitative value" in Scaffold v 3.0, is used to determine relative abundance of proteins within datasets.

### Normalized Spectral Counting and statistical analysis of quantitative proteomics data

Relative abundance levels of identified proteins in healthy and OPML exudates were determined via normalized spectral counting [15], using the quantitative analysis feature in the Scaffold data viewer software (Version 3, Portland, OR). Quantitative values for each protein were compared in the healthy individuals to those in the OPML individuals differences were determined via assigned P-values using the two-tailed students t-test (type 2). All proteins with a P-value of less than 0.05 from the healthy exudate to OPML comparison are included in Additional File 2, Table S1. When screening for inflammation-associated proteins showing differential abundance (Table 1), a P-value threshold of < 0.1 was used.

**Table 1:** Selected proteins showing increased relative abundance in OPML tissue compared to healthy

Protein	Quantitative ratio^1^	P value^1^	Evidence of association with OPML and/or epithelial inflammation
hnRNPM	8.00	0.091	RNA binding, splicing and inflammation signaling; increased abundance of hnRNPs has been measured in OPML tissue ^17^

IL1F6	22.00	0.016	Cytokine involved in inflammation and immune response; increases in abundance in inflamed epithelial tissues ^24^; ^25^

LCN2	4.00	0.021	Iron transporter involved in immune response and apoptosis; activated in inflammatory and pre-malignant tissues ^26^; ^27^

S100A8	2.02	0.088	Calcium binder and pro-inflammatory factor; increases in abundance in OPML tissue ^18^

NQO1	10.00	0.050	Quinone reductase; induced under inflammatory conditions ^28^

XRCC5/6	4.00/8.00	0.016/0.001	Protein complex involved in DNA repair; DNA damage response proteins known to be activated in OPML ^19 ^and other dysplastic epithelial lesions ^29^

^1^Quantitative values and P values determined as described in Experimental Methods

## Results and discussion

Our objective was to determine whether exudate collection from oral lesions coupled with MS-based shotgun proteomics is a viable option for oral cancer biomarker discovery.

To achieve our objective three fundamental questions needed to be answered: 1) Are non-invasively collected tissue exudates compatible with MS-based shotgun proteomics? 2) What is the composition and extent of contamination by saliva of the exudate proteome? 3) Can the differential abundance of protein within exudates collected from different tissue (e.g. healthy tissue vs. OPML) be measured?

**Figure 1:**
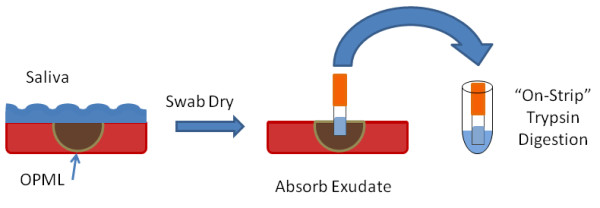
**"On-Strip" digestion method**. In order to produce a peptide solution for MS-based proteomics analysis, we first dried oral epithelium to remove ambient saliva, then placed a PerioPaper strip on the location of interest (Oral Pre-Malignant lesion or normal oral epithelium) and allowed it to absorb exudate. The PerioPaper was next placed in trypsin solution for digestion of proteins to peptides for subsequent processing and mass spectrometry analysis. See Experimental Methods for details.

To answer the first question, we initially explored methods for isolating intact proteins from the PerioPaper strip. For these experiments, we used representative exudate samples collected from healthy oral tissue using the PerioPaper strip as described in Experimental Methods and shown in Figure 1. We first attempted to recover intact proteins from the strips using SDS containing buffers. However, the amount of protein recovered from the strips was very small, at or below the limit of detection for protein quantification using the BCA assay or even reliable detection via SDS-PAGE (data not shown). Given our inability to isolate ample amounts of intact protein, we instead tested an alternate "on-strip" digestion method. We reasoned that direct trypsin digestion of proteins adhered to the PerioPaper strip, without relying on a first step of protein release, may maximize recovery of peptides. Furthermore, a direct "on-strip" digestion would minimize sample handling steps and the use of SDS for protein solubilization, which must be removed prior to MS/MS and can lead to further sample losses. Given these collective advantages, we pursued a simpler and more direct on-strip digestion method. Here, we submerged and boiled the PerioPaper strip in a detergent-free, reducing buffer, added trypsin, and collected liberated peptides after overnight incubation. Initially, we analyzed a 5% aliquot of peptides by MS/MS to evaluate whether adequate amounts of peptides were captured via the on-strip digestion procedure to enable large-scale protein identification. We identified approximately 140 proteins on average from these samples, which compares favorably to the number of identifications generated under identical mass spectrometry analysis and sequence database searching conditions when analyzing complex peptide mixtures of known quantities and loading ≤ 1 ug of total peptides. Therefore we concluded that the remaining 95% of each sample was equivalent to 10-20 μg of protein for the purposes of shotgun MS/MS analysis.

We next employed offline SCX HPLC peptide fractionation [16] in order to increase the number of proteins identified. Using a representative exudate sample, UV absorbance readings at 215 nm and 280 nm were taken during the SCX fractionation. The magnitude of the UV absorbance levels were similar to those measured when fractionating other complex samples of known quantities in the range of 12-15 ug, consistent with our initial estimates above of total peptide amounts based upon the MS/MS results from the 5% aliquots. Fractionation greatly increased our number of protein identifications and sequence coverage for their identifications, producing ~700 identified proteins (< 1% estimated peptide False Discovery Rate).

We then moved on to answering our second question: What is the composition of the OPML proteome and extent of contamination by whole saliva? One initial concern was that the exudate strip would simply absorb saliva, despite our attempts to remove excess ambient salivary fluid from the tissue prior to exudate collection. The high abundant salivary proteins would potentially obscure the identification of lesion-associated proteins. To explore this issue, we compared the protein composition of exudates collected from three different OPML patients, to the composition of whole saliva collected from three different individuals. For this comparison, each whole saliva sample was collected and processed in a similar manner to the exudates, by absorbing saliva onto a PerioPaper strip, followed by on-strip digestion, SCX HPLC fractionation and LC-MS/MS analysis.

**Figure 2:**
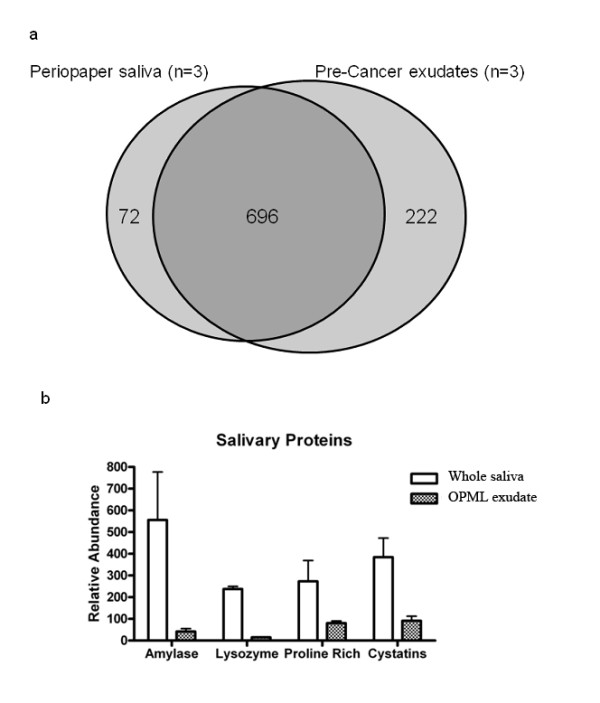
**Exudate proteome is distinct from whole saliva:2a **Venn diagram showing overlap of total protein identifications from PerioPaper collected whole saliva from three individuals compared to OPML exudate proteins from three individuals. **2b **Figure showing the relative proportion of major salivary proteins in OPML exudates compared to brush biopsies. **2a **and **2b**. See Experimental Methods for dataset generation details.

We first compared all proteins identified from three saliva samples to all proteins identified from the OPML exudates (Figure 2a). These results showed that the vast majority of proteins from whole saliva were also identified in the exudate samples, indicating that proteins from whole saliva are still prominent within the exudate samples. Next we focused on some of the highest abundance proteins in whole saliva (salivary amylase, lysozyme, proline-rich proteins, and cystatin proteins). We sought to determine whether the relative amounts of these highly abundant proteins were different between whole saliva and exudates. For this investigation, we used normalized spectral counting as a means to assess the relative abundance of selected high abundance saliva proteins within each sample. As shown in Figure 2b, all of the selected salivary proteins were present in significantly higher relative amounts in the whole saliva samples compared to exudate samples.

**Figure 3:**
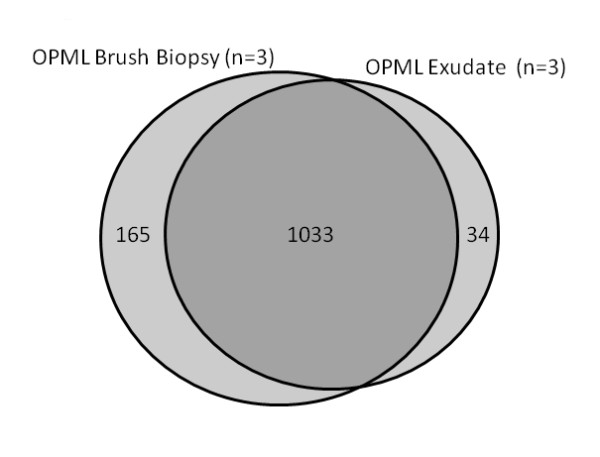
**Exudate proteome is similar to cellular proteome of pre-malignant lesions**. See Experimental Methods for dataset generation details.

To further elucidate the composition of the exudate proteome, we compared it to the proteome derived from the epithelial cells collected from the OPMLs. Here, we collected OPML cells via brush biopsy (see Experimental Methods) from the same three patients that we collected exudates, and analyzed the isolated protein using shotgun proteomics. The proteins identified from the three brush biopsy samples were then compared to the proteins identified from the three exudate samples (Figure 3). The results show that these two sample types are highly similar, with 96% of the proteins found in the exudate samples also present in the brushed cells.

**Figure 4:**
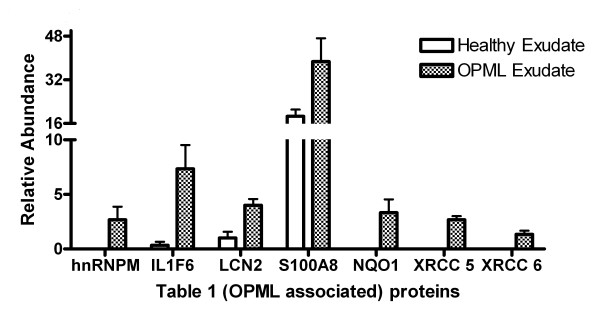
**Plot of abundance levels of inflammation-associated proteins identified in healthy and OPML tissue exudates**. See Table 1 for more details on each protein.

Finally, we sought to answer the question of whether we could measure differential abundance of proteins within exudates collected from different tissue types. Here we decided to compare two distinct tissue types: healthy oral tissue and OPML tissue. Our objective in these experiments was to provide proof-of-principle for conducting quantitative proteomic studies in exudate samples, rather than discover new biomarker candidates. Therefore we focused on expected protein abundance differences within the samples compared that could serve as a benchmark to determine whether we could reliably measure differential protein abundance in exudate samples. Based on prior studies of OPML and similar inflammatory epithelial lesions [17-19], there are numerous proteins which we would expect to show increased abundance within these inflammatory lesions compared to healthy tissues. We analyzed tissue exudates from three different healthy individuals, and three different individuals with OPML. We focused on the numerous proteins showing increased relative abundance in the OPML samples determined via normalized spectral counting and statistical analysis (see Experimental Methods section). Additional File 2, Table S1 shows all proteins determined to show differential abundance between the two groups. Table 1 shows selected proteins with increased relative abundance in the OPML tissue compared to the healthy tissues. As detailed in Table 1, prior studies have established the increased abundance of all of these proteins either in OPMLs or related inflammatory epithelial tissues. Figure 4 graphically shows the measured abundance levels of the proteins in Table 1 as determined via spectral counting.

In this study, we first addressed the fundamental question of whether exudates collected via PerioPaper strips can be analyzed using MS-based proteomics, as new sample types such as these may not contain ample protein amounts to facilitate their analysis using MS. Although isolating intact proteins from the strips proved difficult, our on-strip digestion method liberated ample amounts of peptides for large-scale shotgun proteomic analysis.

We next investigated the composition of the OPML exudate proteome. One concern was absorbance of ambient saliva, and the high abundance proteins contained therein which may obscure the identification of lower abundance exudate proteins. However, comparison of exudate samples to whole saliva samples revealed that high abundance salivary proteins were greatly decreased in their relative amounts within the exudate samples. Thus, our exudate collection procedure sufficiently removes ambient saliva, which should enable identification of proteins sampled directly from the lesion tissue.

Interestingly, the exudate proteome was highly similar to the proteome of epithelial cells collected directly from the lesions via brush biopsy. This indicated that the PerioPaper strip also absorbs cells from the surface of the lesion, whose protein contents can be detected using our processing method. The presence of cellular proteins is in keeping with the accepted definition of an exudate, which is a mixture of fluid, cells and cellular debris on the surface of inflamed tissue [12]. Thus, exudate collection with PerioPaper strips may offer an alternative to brush biopsy collection, with the advantage that the processing of the samples via on-strip trypsin digestion being simpler than processing intact cells collected via brush biopsy, which includes additional steps of cell lysis and protein precipitation. Additionally, a paper-strip based collection method also has potential use in micro- or nano-scale devices for point-of-care clinical testing [20].

Finally we investigated the feasibility of quantitative proteomic analysis of exudate samples collected from different tissue types. By comparing exudates from healthy oral tissue to OPML tissue we expected to identify inflammation-related proteins at increased abundance in the lesion exudates. Indeed this was the case, demonstrated by the proteins listed in Table 1. Thus we conclude that our method of exudate sampling and MS-based proteomic analysis for profiling is compatible with profiling differential protein abundance, necessary for biomarker discovery studies.

## Conclusions

We demonstrate here a promising new method for the non-invasive, direct sampling of oral lesions, compatible with MS-based proteomics. In future studies, we envision application of this method to comparative analysis of OPMLs and malignant oral lesions. This should provide a powerful means to identify protein biomarkers distinguishing these lesion types that may be useful for early detection of malignant transformation. Given our findings, lesion exudates should be amenable to the full suite of proteomic analysis tools, including those aimed at identifying post-translational modifications or sequence variants [21] that may serve as powerful biomarkers of oral cancer. Additionally, exudate samples analyzed by MS-based proteomics should be amenable to a metaproteomics approach [22] seeking to identify bacterial or viral components of oral lesions which may play a role in pathogenesis. Proteins identified within the exudate samples may also serve as a guide to identifying lesion-derived proteins shed into the saliva that could be used for oral cancer detection in this easily collected fluid [6,23]. Finally, the PerioPaper strips could provide the foundation for point-of-care clinical devices for oral cancer diagnostics, given the emergence of such devices designed for paper-based fluid sampling and analysis [20].

## Competing interests

The authors declare that they have no competing interests.

## Authors' contributions

JAK: Conceived experiments, carried out analysis of exudate samples, analyzed and interpreted data, wrote the manuscript. NLR: Conceived experiments, collected samples. CT: carried out analysis of brush biopsy samples, analyzed data. PDJ: Conceived data analysis workflow, analyzed and interpreted data.

BJH: carried out collection and analysis of saliva samples. TJG: Conceived experiments, analyzed and interpreted data, wrote the manuscript. All authors read and approved the final manuscript.

## Supplementary Material

Additional file 1 **Workflow for MS-based proteomic analysis**. Click here for file

Additional file 2 **Proteins with significant differential abundance in healthy tissue versus OPML**. Click here for file
