# Supplementary figures and images for: Evaluating the potential of a novel oral lesion exudate collection method coupled with mass spectrometry-based proteomics for oral cancer biomarker discovery
Source: Clin Proteomics. 2011 Sep 13;8(1):13. doi: 10.1186/1559-0275-8-13 (PMC3200993; doi:10.1186/1559-0275-8-13)

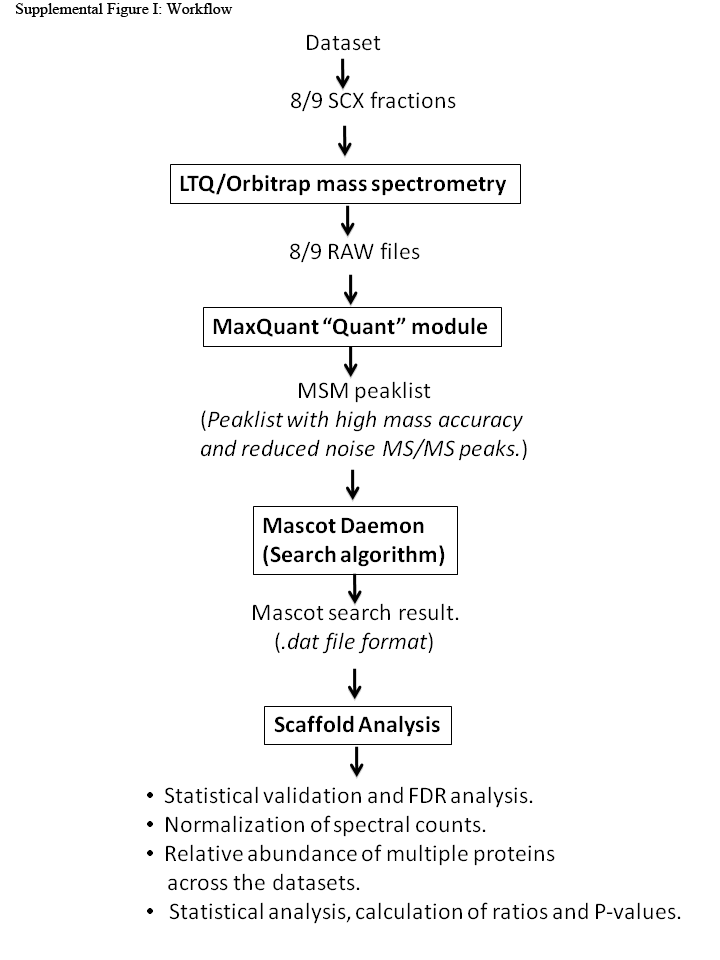

Supplement: Additional file 1 — Workflow for MS-based proteomic analysis. [file 1559-0275-8-13-S1.TIFF]
